# The value of molecular stratification for *CEBPA* ^DM^ and *NPM1* ^MUT^ *FLT3* ^WT^ genotypes in older patients with acute myeloid leukaemia

**DOI:** 10.1111/bjh.13873

**Published:** 2015-12-21

**Authors:** Glenda J. Dickson, Sophia Bustraan, Robert K. Hills, Akbar Ali, Anthony H. Goldstone, Alan K. Burnett, David C. Linch, Rosemary E. Gale

**Affiliations:** ^1^ Department of HaematologyUniversity College London Cancer InstituteLondonUK; ^2^ Department of HaematologyCardiff University School of MedicineCardiffUK; ^3^ Department of HaematologyUniversity College London HospitalLondonUK; ^4^ Present address: Department of Haemato‐OncologyKing's College LondonLondonUK; ^5^ Present address: Faculty of PharmacyNorthern Border UniversityRafhaSaudi Arabia; ^6^ Present address: CTI Life Sciences Ltd.UxbridgeUK

**Keywords:** acute myeloid leukaemia, molecular prognostication, *CEBPA* genotype, *NPM1* and *FLT3* genotype

## Abstract

Older adult patients (≥60 years) with acute myeloid leukaemia (AML) are generally considered to be poor‐risk and there is limited information available regarding risk stratification based on molecular characterization in this age group, particularly for the double‐mutant *CEBPA* (*CEBPA* ^DM^) genotype. To investigate whether a molecular favourable‐risk genotype can be identified, we investigated *CEBPA*,* NPM1* and *FLT3* status and prognostic impact in a cohort of 301 patients aged 60 years or more with intermediate‐risk cytogenetics, all treated intensively. Overall survival (OS) at 1 year was highest in the 12 patients (4%) that were *CEBPA* ^DM^ compared to the 76 (28%) with a mutant *NPM1* and wild‐type *FLT3* (*NPM1* ^MUT^ *FLT3* ^WT^) genotype or all other patients (75%, 54%, 33% respectively), with median survival 15·2, 13·6 and 6·6 months, although the benefit was short‐term (OS at 3 years 17%, 29%, 12% respectively). Combination of the *CEBPA* ^DM^ and *NPM1* ^MUT^ *FLT3* ^WT^ genotype patients defined a molecular group with favourable prognosis (*P *<* *0·0001 in multivariate analysis), with 57% of patients alive at 1 year compared to 33% for all other patients. Knowledge of genotype in older cytogenetically intermediate‐risk patients might influence therapy decisions.

In recent decades there have been considerable improvements in the long‐term outlook for younger adult patients with acute myeloid leukaemia (AML) (Burnett *et al*, 2011). Current therapy is risk‐adapted, based predominantly on cytogenetics and molecular characterization (Dohner *et al*, 2010; Dohner & Gaidzik, 2011; Ofran & Rowe, 2013), and consolidation of first remission by allogeneic transplantation is not usually considered in patients with either good‐risk cytogenetics or a favourable mutation profile, defined as either mutant for nucleophosmin 1 and lacking a fms‐like tyrosine kinase 3 internal tandem duplication (*NPM1* ^MUT^ *FLT3* ^WT^) or double mutant for CCAAT/enhancer binding protein‐α (*CEBPA* ^DM^) (Cornelissen *et al*, 2012; O'Donnell *et al*, 2012). These two mutational categories are almost totally mutually exclusive (Green *et al*, 2010). In our study of younger patients, the presence of an *NPM1* ^MUT^ *FLT3* ^WT^ genotype was associated with a higher complete remission (CR) rate and a lower relapse rate, both contributing to improved survival (Gale *et al*, 2008). The presence of a *CEBPA* ^DM^ genotype was associated with a non‐significantly higher CR rate and a significantly lower relapse rate, leading to improved long‐term survival (Green *et al*, 2010).

However, the median age of AML at diagnosis approximates 70 years (Derolf *et al*, 2009), so that the majority of patients are considered to be elderly (≥60 years), and the improvements seen in the prognosis of younger patients have not been matched by improvements in this older age group (Derolf *et al*, 2009; Burnett *et al*, 2011; Thein *et al*, 2013). This is attributable to both biological factors (e.g. co‐morbidities, poor performance status, pharmacokinetics and pharmacodynamics) and disease‐related factors (e.g. adverse cytogenetic and molecular aberrations, multidrug resistance and antecedent haematological disorders) (Pollyea *et al*, 2011; Ossenkoppele & Lowenberg, 2015).

There is limited information concerning risk stratification in the older compared to younger patients, partly because all older patients have been considered as poor‐risk. The reasons for risk stratification in the older age group, however, are different from those in younger patients. In the older patient fit enough to receive intensive therapy, there is a growing consensus that more intensive therapy, similar to that used in younger patients, results in prolonged survival (Derolf *et al*, 2009; Oran & Weisdorf, 2012), and the quality of life is probably no worse than in those receiving best supportive care or non‐intensive therapy (Alibhai *et al*, 2015). This does not imply that living with AML is not extremely difficult for older patients, and some informed patients might choose not to receive life‐extending therapy. One of the factors to be considered in making this decision is how long patients are likely to live if they elect to receive intensive therapy, and prognostic stratification in the elderly is clearly relevant to this issue.

There is some data suggesting a better outcome, at least in the short‐ to medium‐term, in those older patients with intermediate‐risk (IR) cytogenetics and an *NPM1* ^MUT^ or *NPM1* ^MUT^ *FLT3* ^WT^ genotype, although this largely manifests as increased duration of survival rather than cure (Buchner *et al*, 2009; Becker *et al*, 2010; Lazenby *et al*, 2014), and may be limited to those ≤65 years of age (Ostronoff *et al*, 2015). There is very little data on the impact of *CEBPA* ^DM^ specifically in the older age group. We have therefore determined the impact on survival of the *NPM1*,* FLT3* and *CEBPA* mutation status in a cohort of 301 patients aged 60 years or more with IR cytogenetics who received intensive therapy. We first examined the impact of the presence of a *CEBPA* ^DM^ genotype and then considered the outcome of the combined *NPM1* ^MUT^ *FLT3* ^WT^ and *CEBPA* ^DM^ subgroup (considered as the favourable mutational profile in younger patients) compared to those with any other genotype.

## Methods

### Patients and mutation analysis

Genomic DNA was available from diagnostic samples of 301 (45%) of the 662 patients aged ≥60 years with IR cytogenetics and entered on the UK Medical Research Council (MRC) AML11 trial between 1990 and 1998. Median age was 67 years (range, 60–85). Compared to the 361 patients with IR cytogenetics that were not included in the study, there was no difference in age, sex or type of leukaemia (*de novo*/secondary), CR rate or overall survival (OS), but patients studied were more likely to have a higher presenting white blood cell count (WBC) (Table SI). Ethical approval for the trial was obtained from participating institution's ethics review committees and patients gave informed consent. *FLT3*,* NPM1* and *CEBPA* screening were performed as previously described (Gale *et al*, 2008; Green *et al*, 2010).

### Therapy, clinical endpoints and statistical methods

Details of the trial protocol have been published elsewhere (Goldstone *et al*, 2001). CR was defined as a normocellular bone marrow (BM) containing <5% blasts and showing evidence of normal maturation of other marrow elements. Persistence of myelodysplastic features did not preclude the diagnosis of CR. OS was the time from trial entry to death. For patients achieving CR, relapse‐free survival (RFS) was the time from the date of first CR to an event (death in first CR or relapse) and cumulative incidence of relapse (CIR) was the incidence of relapse after CR, with death in CR as a competing risk.

Mantel‐Haenszel and chi‐squared tests were used to test for differences in demographic and clinical data by genotype. Kaplan–Meier curves were constructed for survival data and compared by means of the log‐rank test. Surviving patients were censored on 9 August, 2010, with follow‐up complete for 98% of patients. Median follow‐up for survival was 16·1 years (range, 13·7–19·5 years). Multivariate Cox models were used to analyse CIR and OS, adjusting for age, secondary leukaemia, WBC, performance status and molecular genotype. Models were fitted using forward selection, with variables added to the model if they had a *P* value, derived using the deviance statistic, of <0·05. Odds ratios (OR) or hazard ratios (HR) and 95% confidence intervals (CI) are quoted for endpoints. In all cases a ratio of <1 indicates benefit. All *P* values are two‐tailed.

## Results

### Patient characteristics according to CEBPA genotype

**Figure 1:**
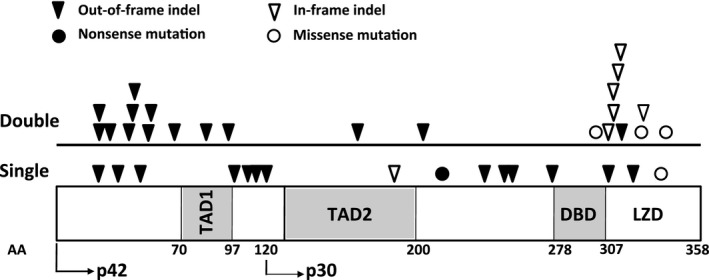
Location and type of mutation detected in *CEBPA*‐single mutant and *CEBPA*‐double mutant patients. Amino acids (AA) encoding the transactivation domains (TAD1 and TAD2), DNA‐binding domain (DBD) and leucine zipper domain (LZD) and the ATG start site for the p30 isoform are indicated.

Details of the cohort studied are shown in Table 1. Overall, 28 patients (9%) were *CEBPA* ^MUT^, 16 (57%; 5% of total cohort) had a single mutation (*CEBPA* ^SM^) and 12 (43%; 4%) were *CEBPA* ^DM^ (Fig 1, Table SII). All *CEBPA* ^DM^ patients had mutations that would be predicted to lead to complete loss of normal C/EBP‐α activity, with N‐terminal mutations leading to production of the p30 isoform or frameshift or nonsense mutations leading to a truncated protein and/or C‐terminal mutations disrupting the DNA binding or leucine zipper domains. There was no significant difference between *CEBPA* ^DM^, *CEBPA* ^SM^ and *CEBPA* ^WT^ patients in age, sex, type of leukaemia, WBC and incidence of either *FLT3* ^ITD^ or *NPM1* ^MUT^, although it should be noted that no patient with a *CEBPA* ^DM^ had the *NPM1* ^MUT^ *FLT3* ^WT^ genotype (*P *=* *0·02) (Table 1).

**Table 1:** Characteristics of the patients studied according to *CEBPA* genotype

Parameter	*CEBPA* ^WT^ (*n* = 273)	*CEBPA* ^SM^ (*n* = 16)	*CEBPA* ^DM^ (*n* = 12)	WT *versus* Single *versus* Double
Age, years				0·19^b^
60–64	92 (34%)	6 (38%)	7 (58%)	
65–69	96 (35%)	5 (31%)	3 (25%)	
≥70	85 (31%)	5 (31%)	2 (17%)	
Median (range)	67 (60–85)	66 (60–75)	63 (60–74)	
Sex				0·5^a^
Female	117 (43%)	8 (50%)	6 (50%)	
Male	156 (57%)	8 (50%)	6 (50%)	
Performance Status				0·8^a^
WHO 0	105 (38%)	8 (50%)	4 (33%)	
WHO 1	121 (44%)	5 (31%)	7 (58%)	
WHO 2	18 (7%)	1 (6%)	0	
WHO 3	23 (8%)	1 (6%)	1 (8%)	
WHO 4	6 (2%)	1 (6%)	0	
Diagnosis				0·5^a^
*De Novo*	202 (74%)	12 (75%)	10 (83%)	
Secondary	71 (26%)	4 (25%)	2 (17%)	
WBC, ×10^9^/l				0·14^b^
0–9·9	90 (33%)	5 (31%)	2 (17%)	
10–49·9	91 (34%)	4 (25%)	6 (50%)	
50–99·9	53 (20%)	3 (19%)	1 (8%)	
≥100	37 (14%)	4 (25%)	3 (25%)	
Median (range)	26·6 (0·3–513·0)	30·5 (1·8–349)	40·2 (4·2–301)	
*FLT3* ^ITD^				0·8^a^
WT	206 (75%)	9 (56%)	11 (92%)	
Mutant	67 (25%)	7 (44%)	1 (8%)	
*NPM1* ^MUT^				0·3^a^
WT	157 (58%)	5 (31%)	11 (92%)	
Mutant	116 (42%)	11 (69%)	1 (8%)	
*NPM1* ^MUT^ *FLT3* ^WT^	76 (28%)	7 (44%)	0 (0%)	0·02^c^
Other	197 (72%)	9 (56%)	12 (100%)	

DM, double mutant; SM, single mutant; ITD, internal tandem duplication; MUT, mutant; WT, wild‐type; WBC, white blood cell count; WHO, World Health Organization.

a Test for trend.

b Spearman correlation.

c Fisher's exact test.

### Response to therapy and outcome of patients with CEBPA mutations

**Figure 2:**
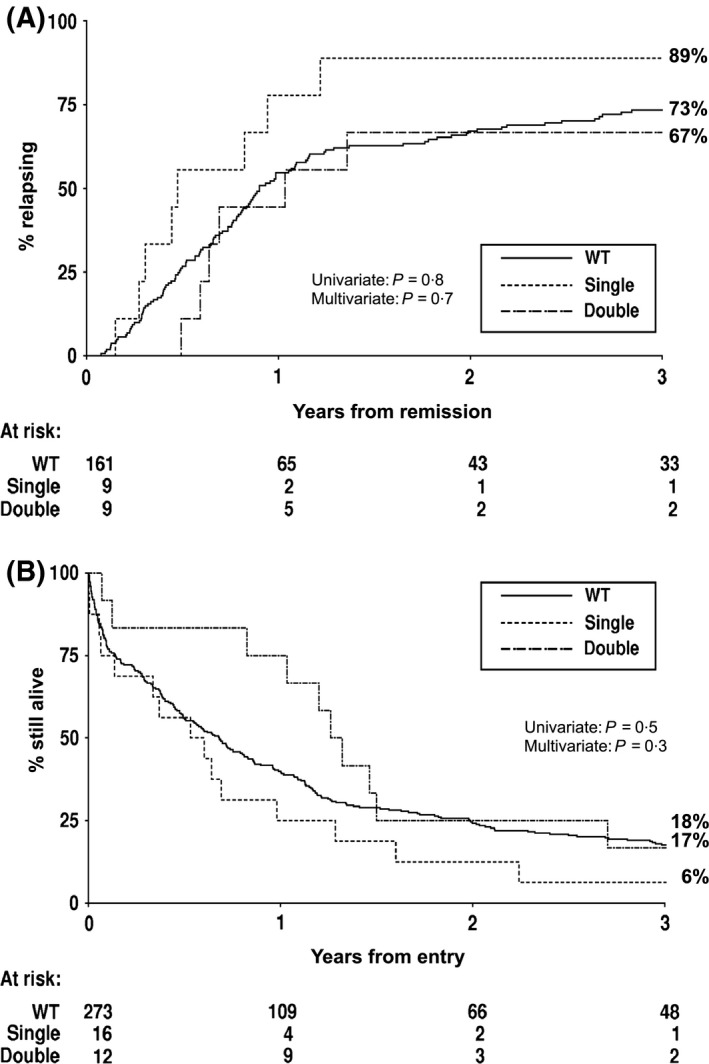
Kaplan–Meier curves stratified according to *CEBPA* genotype. (A) Cumulative incidence of relapse. (B) Overall survival. WT, wild type.

There was no evidence of a benefit in *CEBPA* ^SM^ patients, where response to therapy and outcome were either the same or worse than *CEBPA* ^WT^ patients (Table 2). *CEBPA* ^DM^ patients had a higher CR rate than *CEBPA* ^WT^ patients (75% vs. 59%). Although a relatively large difference, this was not significant in multivariate analysis adjusting for baseline characteristics (OR = 0·33, CI = 0·08–1·38; *P *=* *0·12), which is not unexpected as the number of such cases is small (*n* = 12) (Table 2). CIR was lower in the *CEBPA* ^DM^ patients compared to the *CEBPA* ^WT^ patients, being 44% vs. 55%, respectively, at 1 year and 67% vs. 73% at 3 years (Fig 2A), but again this did not achieve statistical significance (*P *=* *0·4 for *CEBPA* ^DM^ *versus* all others). Short‐term survival was improved for *CEBPA* ^DM^ patients (median 471 d for *CEBPA* ^DM^ and 248 d for *CEBPA* ^WT^), although the benefit was lost by 3 years when the OS was the same (17% vs. 18%) (Fig 2B). In multivariate analysis, there was a trend for a better OS in the *CEBPA* ^DM^ patients when compared to other patients (HR = 0·57, CI = 0·31–1·08; *P *=* *0·08).

**Table 2:** Outcome data according to *CEBPA* genotype

Outcome	*CEBPA* ^WT^ (*n* = 273)	*CEBPA* ^SM^ (*n* = 16)	*CEBPA* ^DM^ (*n* = 12)	*CEBPA* ^WT^ *versus CEBPA* ^SM^ *versus CEBPA* ^DM^ OR or HR (95% CI), *P*‐value		*CEBPA* ^DM^ *versus* not OR or HR (95% CI), *P*‐value	
				Univariate	aMultivariate	Univariate	aMultivariate
CR/CRi	59%	56%	75%	0·79 (0·46–1·37), *P *=* *0·4	0·71 (0·39–1·27), *P *=* *0·2	0·51 (0·16–1·66), *P *=* *0·3	0·33 (0·08–1·38), *P *=* *0·12
30‐d mortality	20%	25%	8%	0·82 (0·42–1·57), *P *=* *0·5	0·76 (0·38–1·51), *P *=* *0·4	0·53 (0·15–1·84), *P *=* *0·3	0·35 (0·05–2·60), *P *=* *0·3
3‐year OS	18%	6%	17%	0·92 (0·71–1·19), *P *=* *0·5	0·87 (0·67–1·13), *P *=* *0·3	0·75 (0·45–1·27), *P *=* *0·3	0·57 (0·31–1·08), *P *=* *0·08
3‐year RFS	21%	11%	22%	0·95 (0·69–1·31), *P *=* *0·8	0·93 (0·68–1·29), *P *=* *0·7	0·83 (0·43–1·59), *P *=* *0·6	0·74 (0·36–1·55), *P *=* *0·4
3‐year CIR	73%	89%	67%	0·96 (0·68–1·35), *P *=* *0·8	0·94 (0·66–1·34), *P *=* *0·7	0·75 (0·37–1·52), *P *=* *0·4	0·68 (0·29–1·58), *P *=* *0·4

95% CI, 95% confidence intervals; CIR, cumulative incidence of relapse; CR, complete remission; CRi, complete remission with incomplete haematological recovery; DM, double mutant; HR, hazard ratio; OR, odds ratio; OS, overall survival; RFS, relapse‐free survival; SM, single mutant; WT, wild‐type.

a Adjusted for age, secondary leukaemia, white blood cell count, performance status, *FLT3* and *NPM1* genotype.

### Comparison of response to therapy and outcome in patients with CEBPA^DM^, NPM1^MUT^FLT3^WT^ and Other genotypes

**Figure 3:**
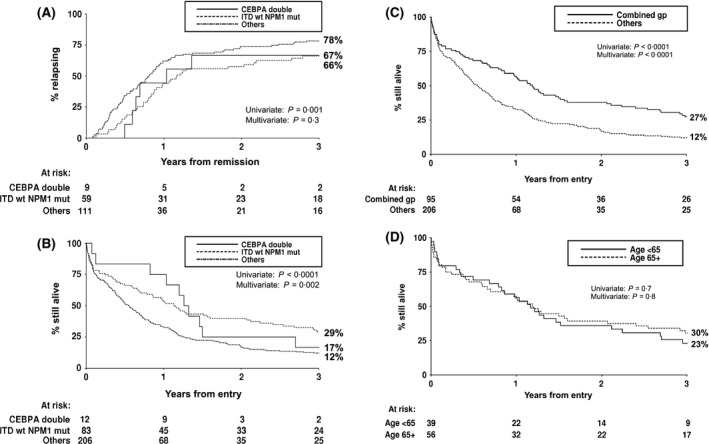
Kaplan–Meier curves stratified according to *CEBPA* ^DM^ and *NPM1* ^MUT^ *FLT3* ^WT^ genotype. (A) Cumulative incidence of relapse and (B) overall survival in the three genotype groups. (C) Overall survival for the combined favourable‐risk *CEBPA* ^DM^ and *NPM1* ^MUT^ *FLT3* ^WT^ group compared with all other patients. (D) Overall survival stratified according to age in the favourable‐risk group. CEBPA double, *CEBPA* ^DM^ genotype; ITD wt NPM1 mut, *NPM1* ^MUT^ *FLT3* ^WT^ genotype.

Although the *CEBPA* ^DM^ genotype appeared to be associated with only a limited benefit in outcome, the above analysis is likely to be influenced by the presence of good‐risk *NPM1* ^MUT^ *FLT3* ^WT^ patients in the non‐*CEBPA* ^DM^ group, which would attenuate any difference between *CEBPA* ^DM^ patients and those with a poor‐risk genotype. Overall, 83 patients (28%) had an *NPM1* ^MUT^ *FLT3* ^WT^ genotype and it was mutually exclusive with a *CEBPA* ^DM^ genotype. We therefore divided the patients into three groups, *NPM1* ^MUT^ *FLT3* ^WT^ patients, *CEBPA* ^DM^ patients, and all other patients (i.e. those with an *NPM1* ^WT^ or *NPM1* ^MUT^ *FLT3* ^ITD^ genotype), hereafter called ‘Other’ genotypes. The CR rate in patients with an *NPM1* ^MUT^ *FLT3* ^WT^ genotype was 71% compared to 75% in the *CEBPA* ^DM^ patients and 54% in those with ‘Other’ genotypes (Table 3). *NPM1* ^*MUT*^ *FLT3* ^*WT*^ or *CEBPA* ^DM^ patients had a lower CIR at 1 year than the remaining patients (44%, 56% and 62% respectively). However, this difference was less apparent by 3 years (66%, 67% and 78% respectively) and, although it was statistically significant in univariate analysis (*P *=* *0·01 for the 3‐way comparison), it did not retain significance after adjustment for other factors (*P *=* *0·3) (Fig 3A). Survival at 1 year was highest in the *CEBPA* ^DM^ group, but this then fell towards the level of the patients in the ‘Other’ genotype category and showed no difference by 2 years (Fig 3B). Even so, multivariate analysis showed that the OS was significantly better in the *CEBPA* ^DM^ patients than in the ‘Other’ genotypic group (HR = 0·52, CI = 0·28–0·97, *P *=* *0·04). Similarly, OS was significantly better in the *NPM1* ^MUT^ *FLT3* ^WT^ group compared to the ‘Other’ genotype group (*P *<* *0·0001 for univariate analysis; *P *=* *0·002 for multivariate analysis). Median survival was 13·6, 15·2 and 6·6 months, respectively, in the *NPM1* ^MUT^ *FLT3* ^WT^, *CEBPA* ^DM^ and ‘Other’ groups.

**Table 3:** Outcome data comparing the *CEBPA* ^DM^ and *NPM1* ^MUT^ *FLT3* ^WT^ favourable‐risk groups

Outcome	*CEBPA* ^DM^ (*n* = 12)	*NPM1* ^MUT^ *FLT3* ^WT^ (*n* = 83)	Others (*n* = 206)	*CEBPA* ^DM^ *versus NPM1* ^MUT^ *FLT3* ^WT^		*CEBPA* ^DM^ *versus* Others		*NPM1* ^MUT^ *FLT3* ^WT^ *versus* Others	
				Univariate	aMultivariate	Univariate	aMultivariate	Univariate	aMultivariate
CR/CRi	75%	71%	54%						
OR (95% CI)				0·84 (0·22–3·14)	0·83 (0·20–3·57)	0·43 (0·13–1·37)	0·39 (0·10–1·57)	0·43 (0·13–1·37)	0·39 (0·10–1·57)
*P*				0·8	0·8	0·15	0·18	0·15	0·18
30‐d mortality	8%	18%	21%						
HR (95% CI)				0·53 (0·13–2·23)	0·33 (0·04–2·70)	0·51 (0·15–1·79)	0·33 (0·05–2·46)	0·51 (0·15–2·79)	0·33 (0·05–2·46)
*P*				0·4	0·3	0·3	0·3	0·3	0·3
OS									
1 year	75%	54%	33%						
3 years	17%	29%	12%						
HR (95% CI)				1·10 (0·57–2·12)	0·93 (0·47–1·84)	0·64 (0·40–1·04)	0·52 (0·28–0·97)	0·64 (0·40–1·04)	0·52 (0·28–0·97)
*P*				0·8	0·8	0·07	0·04	0·07	0·04
RFS									
3 years	22%	31%	14%						
HR (95% CI)				1·18 (0·53–2·61)	1·04 (0·47–2·30)	0·70 (0·38–1·27)	0·66 (0·32–1·37)	0·70 (0·38–1·27)	0·66 (0·32–1·37)
*P*				0·7	0·9	0·2	0·3	0·2	0·3
CIR									
1 year	56%	44%	62%						
3 years	67%	66%	78%						
HR (95% CI)				1·01 (0·42–2·42)	0·84 (0·34–2·06)	0·65 (0·34–1·27)	0·59 (0·25–1·36)	0·65 (0·34–1·27)	0·59 (0·25–1·36)
*P*				1·0	0·7	0·2	0·2	0·2	0·2

95% CI, 95% confidence intervals; CIR, cumulative incidence of relapse; CR, complete remission; CRi, complete remission with incomplete haematological recovery; DM, double mutant; HR, hazard ratio; MUT, mutant; OR, odds ratio; OS, overall survival; RFS, relapse‐free survival; WT, wild‐type.

a Adjusted for age, secondary leukaemia, white blood cell count and performance status.

As outcome was broadly comparable for the *CEBPA* ^DM^ and *NPM1* ^MUT^ *FLT3* ^WT^ patients, they were combined into a favourable‐risk group, as in younger patients, together comprising 32% of the patients in this study. OS was very significantly better in this combined group than the ‘Other’ genotypes group, 57% vs. 33% at 1 year, and 27% vs. 12% at 3 years (HR = 0·50, CI = 0·38–0·65, *P *<* *0·0001 in multivariate analysis) (Fig 3C). Median survival was 14·3 months in the favourable‐risk group compared to only 6·6 months in the remainder. There was no evidence that survival in the favourable‐risk group differed according to age: OS at 3 years was 30% for the 56 patients (59%) aged >65 years compared to 23% for those aged <65 years (HR = 0·92, CI = 0·59–1·44; *P *=* *0·7) (Fig 3D), median survival 14·3 and 14·0 months, respectively.

## Discussion

In younger adult AML patients the presence of a *CEBPA* ^DM^ genotype is associated with better response to treatment and improved long‐term outcome (Green *et al*, 2010; Taskesen *et al*, 2011). It is sometimes assumed that this is also true in older patients (Ossenkoppele & Lowenberg, 2015), but this has never been formally demonstrated, with such results only presented within a much wider age range of patients (Renneville *et al*, 2009; Wouters *et al*, 2009; Dufour *et al*, 2010; Fasan *et al*, 2014). The study presented here is the first to report on the impact of a *CEBPA* ^DM^ genotype specifically in patients ≥60 years of age. The incidence of 4% *CEBPA* ^DM^ in the present cohort was similar to the 5% incidence reported in our study of younger patients with IR cytogenetics (Green *et al*, 2010), and is consistent with other studies where age and double/single mutant status have been given (Dufour *et al*, 2010; Marcucci *et al*, 2012). A *CEBPA* ^DM^ genotype was associated with improved short‐term survival that was significant in multivariate analysis when compared to patients without either a *CEBPA* ^DM^ or *NPM1* ^MUT^ *FLT3* ^WT^ genotype. It is clear that this improvement is relatively short‐lived and does not equate to a high cure rate.

Older patients with an *NPM1* ^MUT^ *FLT3* ^WT^ genotype have already been shown to have an improved 1‐year OS (Buchner *et al*, 2009; Lazenby *et al*, 2014; Ostronoff *et al*, 2015), and this was confirmed in the present cohort. The combined favourable‐risk genotypic group reported here, of either a *CEBPA* ^DM^ or an *NPM1* ^MUT^ *FLT3* ^WT^ genotype, accounted for 32% of all the patients investigated, although it must be acknowledged that this cohort was restricted to patients deemed fit enough to receive intensive chemotherapy. Even in this favourable‐risk genotypic group only 57% of the patients were alive at 1 year and the corollary of identifying a favourable group is that, by default, an unfavourable‐risk group is also identified. In this cohort of patients, of those without a favourable‐risk genotype, nearly 50% had died by 6 months and only 12% were alive at 3 years.

Although the cohort presented here was treated two decades ago, there has been very little progress in the intervening years in improving outcome in this age group and the findings are still likely to apply. This disease‐related information needs to be integrated with other patient‐related information, including co‐morbidities, but for some patients it may influence the decision of whether or not to receive intensive therapy. Therefore consideration should be given to offering molecular screening as part of the diagnostic work‐up for older patients with IR cytogenetics. Nevertheless, even for the more favourable‐risk group, there remains an undoubted need to develop novel therapeutic strategies for older patients with AML.

## Author contributions

Glenda J. Dickson performed research, analysed data and wrote the paper. Sophia Bustraan and Akbar Ali performed research. Robert K. Hills performed statistical analysis. Anthony H. Goldstone and Alan K. Burnett were the chief investigators of the trial. David C. Linch designed the study and wrote the paper. Rosemary E. Gale designed the study, analysed data and wrote the paper. All authors approved the final version of the manuscript.

## Conflicts of interest

The authors have no competing conflicts of interest to declare.

## Supporting information


**Table SI.** Characteristics of UK AML11 trial patients aged ≥60 years and with IR cytogenetics that were excluded and included in the molecular investigation.
**Table SII.** Details of the mutations identified in the *CEBPA* ^MUT^ cases.
