# Supplementary material for: The value of molecular stratification for CEBPA DM and NPM1 MUT FLT3 WT genotypes in older patients with acute myeloid leukaemia
Source: Br J Haematol. 2015 Dec 21;172(4):573–80. doi: 10.1111/bjh.13873 (PMC4855634; doi:10.1111/bjh.13873)
Supplement: Supplementary file 1 — Table SI. Characteristics of UK AML11 trial patients aged ≥60 years and with IR cytogenetics that were excluded and included in the molecular investigation. Table SII. Details of the mutations identified in the CEBPA MUT cases. [file BJH-172-573-s001.docx]

Supplementary Table 1. Characteristics of UK AML11 trial patients aged ≥60 years and with IR cytogenetics that were excluded and included in the molecular investigation.

| Parameter | Not included (n=361) | Included (n=301) | *P* value |
| --- | --- | --- | --- |
| Age, Years:  60-64  65-69  ≥70  Median (range) | 101 (28%)  123 (34%)  137 (38%)  67 (60-91) | 105 (35%)  104 (35%)  92 (31%)  67 (60-85) | 0.2** |
| Sex:  Female  Male | 153 (42%)  208 (58%) | 131 (44%)  170 (56%) | 0.8 |
| Performance Status:  WHO 0  WHO 1  WHO 2  WHO 3  WHO 4 | 182 (50%)  122 (34%)  31 (9%)  24 (7%)  2 (1%) | 117 (39%)  133 (44%)  19 (6%)  25 (8%)  7 (2%) | 0.02* |
| Diagnosis:  De Novo  Secondary | 290 (80%)  71 (20%) | 224 (74%)  77 (26%) | 0.07 |
| WBC, x10^9^/l:  0-9.9  10-49.9  50-99.9  ≥100  Median (range) | 186 (52%)  88 (25%)  40 (11%)  41 (12%)  8.4 (0.3-461.0) | 97 (32%)  101 (34%)  57 (19%)  44 (15%)  27.4 (0.3-513.0) | <.0001** |
| Transplanted:  No  Yes | 358 (99%)  3 (1%) | 299 (99%)  2 (1%) | 1.0*** |
| CR rate | 60% | 59% | 1.0† |
| OS at 3 years | 17% | 17% | 0.5‡ |

*Mantel-Haenszel test for trend; **Wilcoxon rank sum test; ***Fisher exact test; †Mantel-Haenszel test; ‡Log-rank test

Abbreviations: CR, complete remission; OS, overall survival; WBC, white blood cell count

Supplementary Table 2. Details of the mutations identified in the *CEBPA*^MUT^ cases.

| **No.** | **DNA change 1** | **Predicted AA change 1** | **DNA change 2** | **Predicted AA change 2** |
| --- | --- | --- | --- | --- |
| Single mutant cases | | | | |
| 1 | c.68dupC | p.H24fs |  |  |
| 2 | c.102_103delinsT | p.G35fs |  |  |
| 3 | c.135dupA | p.P46fs |  |  |
| 4 | c.287_311del | p.P97fs |  |  |
| 5 | c.317_318insGT | p.F106fs |  |  |
| 6 | c.347delG | p.G116fs |  |  |
| 7 | c.358dupA | p.M120fs |  |  |
| 8 | c.558_566del | p.P187_P189del |  |  |
| 9 | c.643C>T | p.Q215X |  |  |
| 10 | c.709_710delinsGCGG | p.P237fs |  |  |
| 11 | c.741_759del | p.G248fs |  |  |
| 12 | c.750delC | p.S251fs |  |  |
| 13 | c.822dupC | p.K275fs |  |  |
| 14 | c.914delA | p.Q305fs |  |  |
| 15 | c.962dupA | p.N321fs |  |  |
| 16 | c.1009A>T | p.T337S |  |  |
| Double mutant cases | | | | |
| 17 | c.68dupC | p.H24fs | c.925_927del | p.E109del |
| 18 | c.68dupC | p.H24fs | c.247delC | p.Q83fs |
| 19 | c.85dupG | p.A29fs | c.938_939insTA | p.K313fs |
| 20 | c.117dupC | p.A40fs | c.896G>T | p.S299I |
| 21 | c.125_138del | p.P42fs | c.925_926delinsACGGTTCC | p.E309delinsTVP |
| 22 | c.129dupC | p.A44fs | c.927_974dup | p.R325_K326ins16 |
| 23 | c.147_165del | p.E50fs | c.934_936dup | p.Q312dup |
| 24 | c.153_187delinsT | p.L52fs | c.934_935insTCC | p.P311_Q312insL |
| 25 | c.185_188dup | p.I64fs | c.498_499insA | p.E167fs |
| 26 | c.282_291del | p.V95fs | c.914_916dup | p.Q305_R306insQ |
| 27 | c.611delC | p.P204fs | c.971T>C | p.L324P |
| 28 | c.922T>A[HOM] | p.L331Q |  |  |

Nucleotides numbered from the major translational start codon at nucleotide position 1.

Abbreviations: AA, amino acid; del, deletion; dup, duplication; fs, frame-shift; HOM, homozygous mutation; ins, insertion.
